# Deficient glutamate biosynthesis triggers a concerted upregulation of ribosomal protein genes in Arabidopsis

**DOI:** 10.1038/s41598-017-06335-4

**Published:** 2017-07-21

**Authors:** Tamara Muñoz-Nortes, José Manuel Pérez-Pérez, Raquel Sarmiento-Mañús, Héctor Candela, José Luis Micol

**Affiliations:** Instituto de Bioingeniería, Universidad Miguel Hernández, Campus de Elche, 03202 Elche, Spain

## Abstract

Biomass production requires the coordination between growth and metabolism. In a large-scale screen for mutants affected in leaf morphology, we isolated the *orbiculata1* (*orb1*) mutants, which exhibit a pale green phenotype and reduced growth. The combination of map-based cloning and next-generation sequencing allowed us to establish that *ORB1* encodes the GLUTAMATE SYNTHASE 1 (GLU1) enzyme, also known as FERREDOXIN-DEPENDENT GLUTAMINE OXOGLUTARATE AMINOTRANSFERASE 1 (Fd-GOGAT1). We performed an RNA-seq analysis to identify global gene expression changes in the *orb1–3* mutant. We found altered expression levels of genes encoding enzymes involved in nitrogen assimilation and amino acid biosynthesis, such as glutamine synthetases, asparagine synthetases and glutamate dehydrogenases, showing that the expression of these genes depends on the levels of glutamine and/or glutamate. In addition, we observed a concerted upregulation of genes encoding subunits of the cytosolic ribosome. A gene ontology (GO) analysis of the differentially expressed genes between L*er* and *orb1–3* showed that the most enriched GO terms were ‘translation’, ‘cytosolic ribosome’ and ‘structural constituent of ribosome’. The upregulation of ribosome-related functions might reflect an attempt to keep protein synthesis at optimal levels even when the pool of glutamate is reduced.

## Introduction

The final shape and size of leaves depends on a complex sequence of developmental events, which include the recruitment of cells to the incipient leaf primordium, the control of cell proliferation, the transition from cell proliferation to cell expansion, and cell expansion and differentiation^1–3^. Leaf size also depends on the availability, absorption and assimilation of nutrients, mainly nitrogen, whose metabolism has to be tightly coordinated with carbon metabolism to promote biomass accumulation^4–7^. Because plant biomass is the outcome of interactions between metabolism and growth, understanding how metabolic pathways supply the building blocks for the growth of developing plant organs is a fundamental step towards the goal of engineering more productive crops with increased biomass^8^. Previous studies in *Arabidopsis thaliana* (hereafter, Arabidopsis) and other plant species have shown that the production of biomass can be enhanced by manipulating the expression of genes that encode positive and negative regulators of cell proliferation and expansion^9–16^. In Arabidopsis, a positive effect on plant growth and biomass accumulation occurs when certain genes are overexpressed. Two examples are *GROWTH-REGULATING FACTOR*5 (*GRF5*) and *EXPANSIN10* (*EXP10*), which respectively encode a transcription factor and an expansin^9–11^. By contrast, other genes function as negative regulators of plant growth. Loss of function mutations of the *BIG BROTHER* (*BB*) and *DA2* genes, which encode E3 ubiquitin ligases, enhance cell proliferation^15, 16^. As an alternative approach, modifying the expression levels of key enzymes in primary carbon and nitrogen metabolic pathways might also lead to increased biomass production^17^.

In the leaves, light harvesting and carbon fixation occur in the chloroplasts, where the biosynthesis of essential metabolites required for the rapid growth of developing tissues takes place^18^. The dual role of leaves as both active metabolic sources and sinks makes them very sensitive to mutations that damage central biosynthetic pathways, often leading to plants with reduced growth. Indeed, many genes identified in an ethyl methanesulfonate (EMS) screen for leaf developmental mutants have been found to encode enzymes that catalyze steps of diverse metabolic pathways and other housekeeping functions^19–26^. The *VENOSA3* (*VEN3*) and *VEN6* genes of Arabidopsis encode the two subunits of the carbamoyl phosphate synthetase, which catalyzes the conversion of glutamine and bicarbonate into carbamoyl phosphate and glutamate in the arginine biosynthesis pathway^21^. *VEN1*, also known as *WEAK ETHYLENE INSENSITIVE2*/*ANTHRANILATE SYNTHASE α1* (*WEI2*/*ASA1*), encodes the α subunit of anthranilate synthase, which catalyzes the conversion of chorismate to anthranilate, the rate-limiting step in the tryptophan biosynthesis pathway^24^. *RUGOSA1* (*RUG1*) encodes the porphobilinogen deaminase, also known as hydroxymethylbilane synthase, which catalyzes the deamination and polymerization of four molecules of porphobilinogen into the linear tetrapyrrole 1-hydroxymethylbilane, the fifth step of tetrapyrrole biosynthesis^22^. The *EXIGUA1* (*EXI1*), *EXI2* and *EXI5* genes encode the CELLULOSE SYNTHASE 8 (CESA8), CESA7 and CESA4 catalytic subunits of the cellulose synthase complex, which is required for secondary cell wall synthesis^23^. In addition, many other genes identified in this screen, such as *APICULATA2* (*API2*), *ANGUSTA3* (*ANG3*), *DENTICULATA5* (*DEN5*), *DEN12*, *DEN29* and *DEN30*, encode different subunits of the cytosolic ribosome, highlighting the close association between active cell proliferation and protein synthesis during leaf development^19, 20, 25^.

In this work, we focus on the *orbiculata1* (*orb1*) mutants, which were isolated in the screen mentioned above. *orb1* mutants have small, round, pale green leaves with no apparent patterning defects. We have found *ORB1* to be the same gene as At5g04140, also known as *GLUTAMATE SYNTHASE 1* (*GLU1*) and *FERREDOXIN-DEPENDENT GLUTAMINE OXOGLUTARATE AMINOTRANSFERASE 1* (*Fd-GOGAT1*). The Fd-GOGAT1 enzyme catalyzes the synthesis of glutamate from glutamine and α-ketoglutarate. Together with the conversion of glutamate and ammonium into glutamine using ATP, which is catalyzed by GLUTAMINE SYNTHETASE 2 (GS2), this reaction is an essential component of the GS/GOGAT cycle. The GS/GOGAT cycle plays a key role in the primary assimilation of exogenous ammonium^27^, and in the re-assimilation of the ammonium released during photorespiration^28^. Glutamate is the major amino-group donor for the biosynthesis of many different amino acids and other nitrogen-containing compounds^29^. Tobacco mutants lacking Fd-GOGAT1, which do not produce glutamate, have been reported to have altered levels of other amino acids^28^. Previous studies have found a significant correlation between glutamate content and shoot biomass in barley, as well as between glutamate content and high productivity in rice, as expected if this amino acid acts as a metabolic hub linking numerous biosynthetic pathways with growth and development^30–32^. Our RNA-seq profiling of *orb1* mutant leaves has given insight on how nitrogen-related metabolic pathways are regulated during the vegetative phase, and shows some differences with the results of a microarray analysis previously performed using a different allele of the same gene^33^. In addition, our analysis of RNA-seq results has unveiled a concerted transcriptional increase of genes encoding components of the translational machinery in *orb1* mutants.

## Results and Discussion

### *orb1* mutants have small, pale green leaves

**Figure 1:**
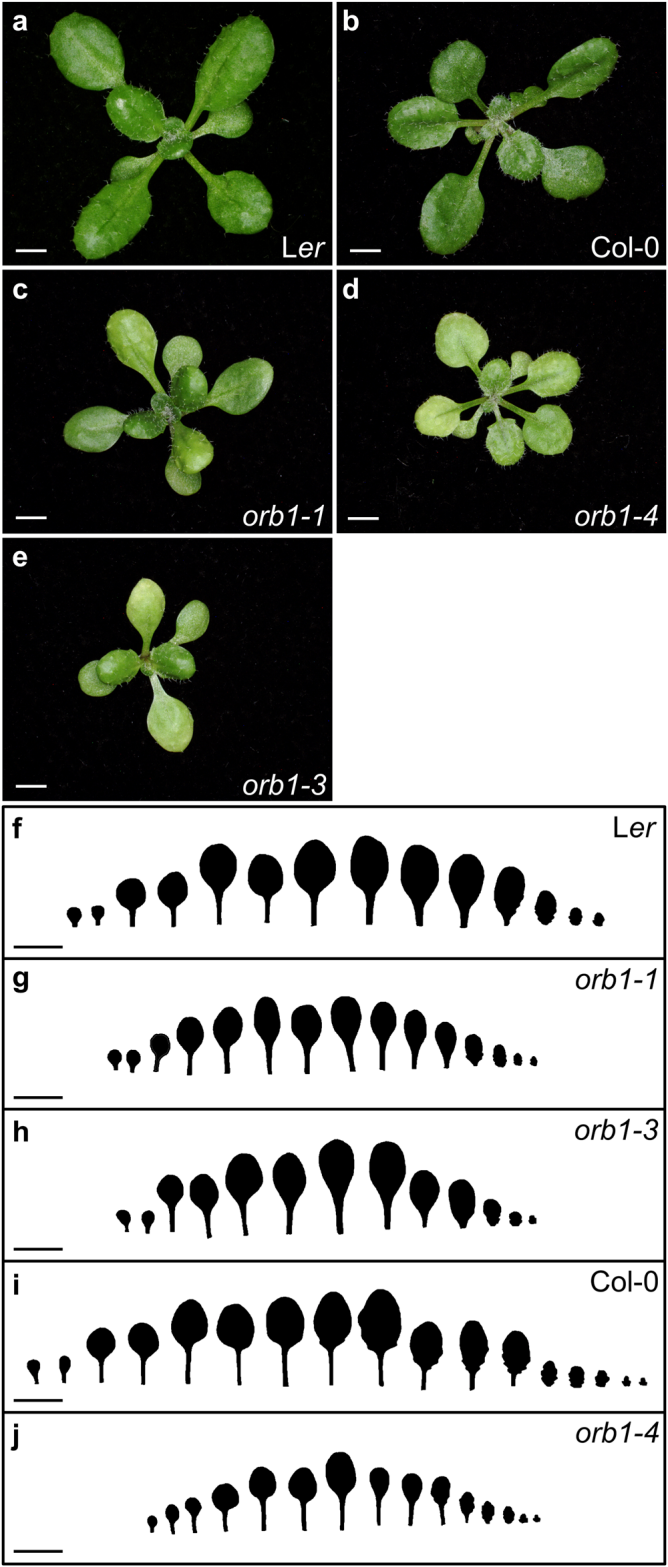
Rosette and leaf shape of *orb1* mutants. The *orb1-1* and *orb1-3* mutants are in a L*er* genetic background, and that of *orb1-4* is Col-0. (**a–e**) Rosette pictures from *orb1* mutants. (**f–j**) Drawings of leaves from *orb1* mutants. Plants were collected **(a–e**) 16 and (f-j) 21 days after stratification (das). Scale bars indicate (**a–e**) 2 mm, and **(f–j**) 1 cm.

**Figure 2:**
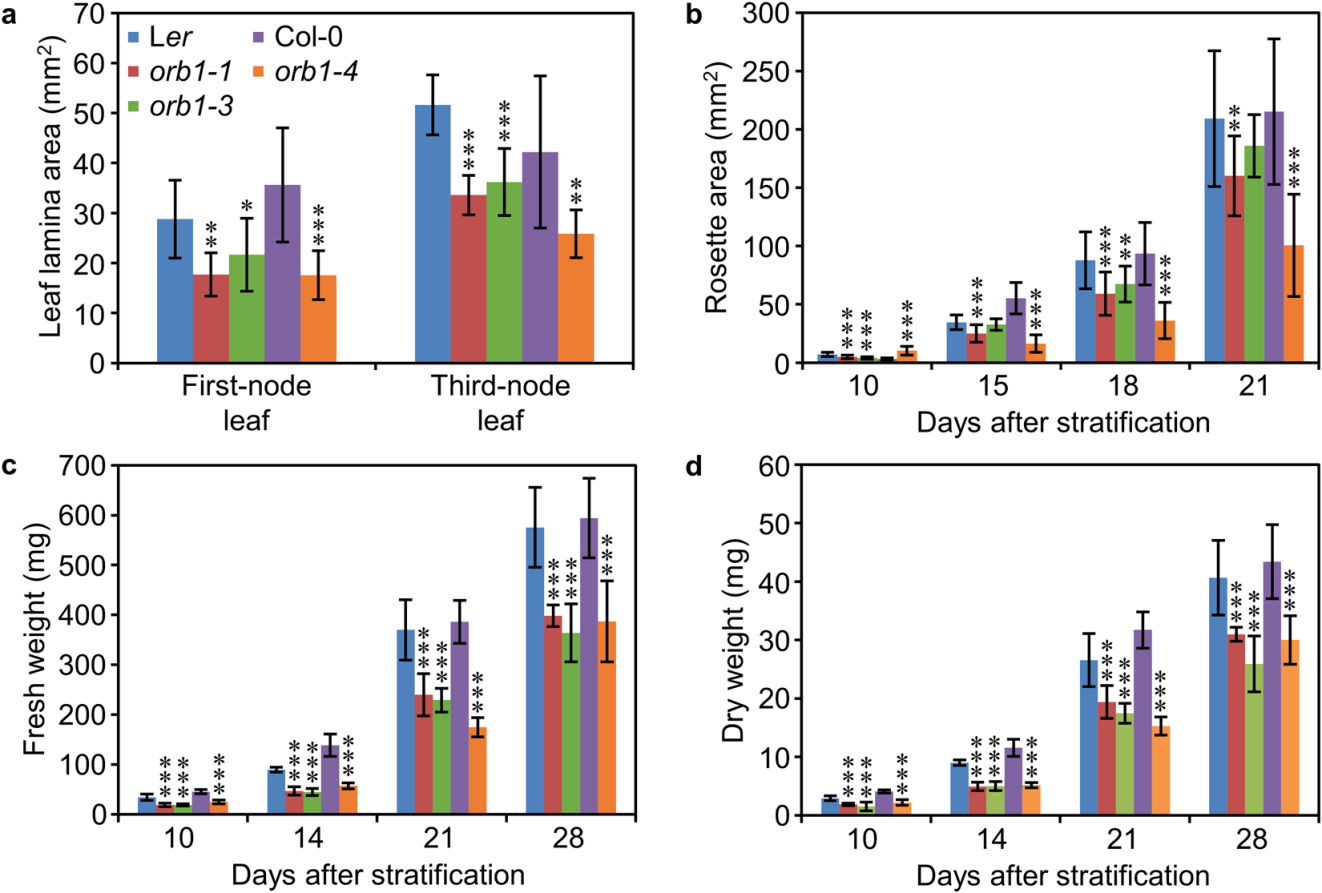
Size and mass of *orb1* mutants. (**a**) Area of the lamina of first- and third-node leaves, (**b**) rosette area, (**c,d**) whole plant (**c**) fresh weight and (**d**) dry weight of L*er*, *orb1-1*, *orb1-3*, Col-0 and *orb1-4* plants. Error bars indicate standard deviations. Asterisks indicate values significantly different from the corresponding wild type in (**a,c,d**) a Mann-Whitney U-test (***p < 0.001, **p < 0.01, *p < 0.05, n = 8–10), and (**b**) a Student’s *t*-test (***p < 0.001, **p < 0.01, n = 14–30).

In order to identify genes involved in leaf development, a large-scale screen for EMS-induced mutants of Arabidopsis with abnormal leaf growth or pigmentation was performed in the laboratory of J.L. Micol, which led to the isolation of the allelic *orbiculata1–1* (*orb1-1*), *orb1-2* and *orb1-3* mutants, among many others^26^. An additional, loss-of-function allele (*orb1-4*) was identified in the SALK collection of T-DNA insertional mutants (SALK_011035 C). The *orb1* mutations are recessive and cause a similar phenotype, including reduced leaf growth and pale green pigmentation (Fig. 1a–e). We have characterized the *orb1-1*, *orb1-3* and *orb1-4* mutants in more detail. Our measurements of first- and third-node leaves collected 21 days after stratification (das) uncovered a significant reduction in the area of the leaf lamina of the *orb1-1*, *orb1-3* and *orb1-4* mutants (p < 0.05; Fig. 2a). In line with this reduction, the basal rosettes of *orb1-1*, *orb1-3* and *orb1-4* were significantly smaller than those of their wild types, the Landsberg *erecta* (L*er*) and Columbia-0 (Col-0) accessions. This growth defect was most severe in the *orb1-4* mutant, whose rosette area was significantly reduced compared to Col-0 (p < 0.001; n = 14-30; Fig. 2b). The projected area of *orb1-1* and *orb1-3* rosettes was also significantly smaller than that of L*er* rosettes (p < 0.01; n = 14–30; Fig. 2b). In addition, the *orb1–1*, *orb1-3* and *orb1-4* mutants exhibited reductions in their fresh and dry weights, which were statistically significant throughout plant development (p < 0.001; n = 8; Fig. 2c,d).

**Figure 3:**
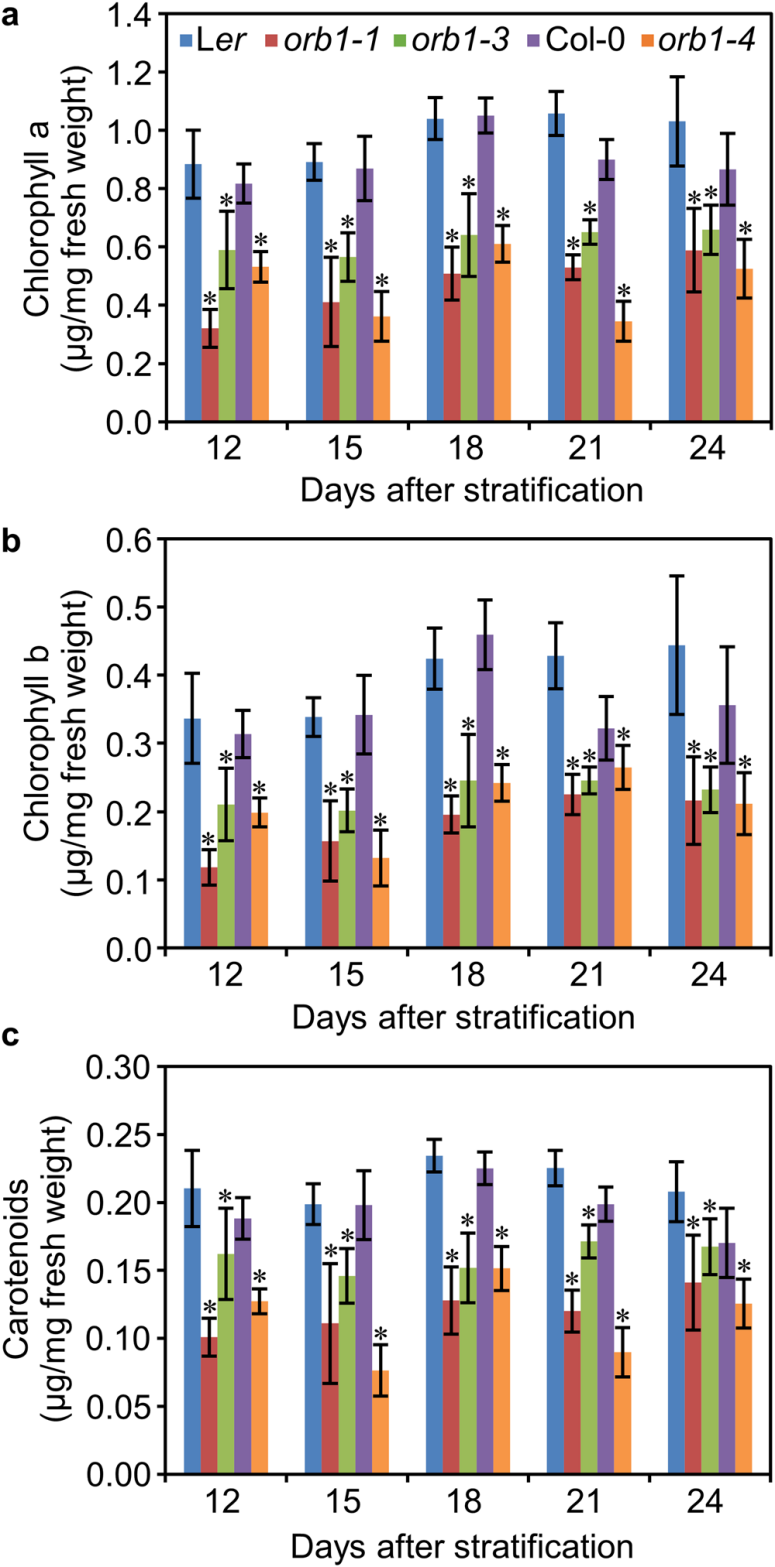
Pigment content in *orb1* mutants. Concentration of (**a**) chlorophyll a, (**b**) chlorophyll b, and (**c**) carotenoids in L*er*, *orb1-1*, *orb1-3*, Col-0 and *orb1-4* above-ground tissues. Plants were collected 12, 15, 18, 21 and 24 das. Error bars indicate standard deviations. Asterisks indicate values significantly different from the corresponding wild type in a Mann-Whitney U-test (*p < 0.05, n = 5).

**Figure 4:**
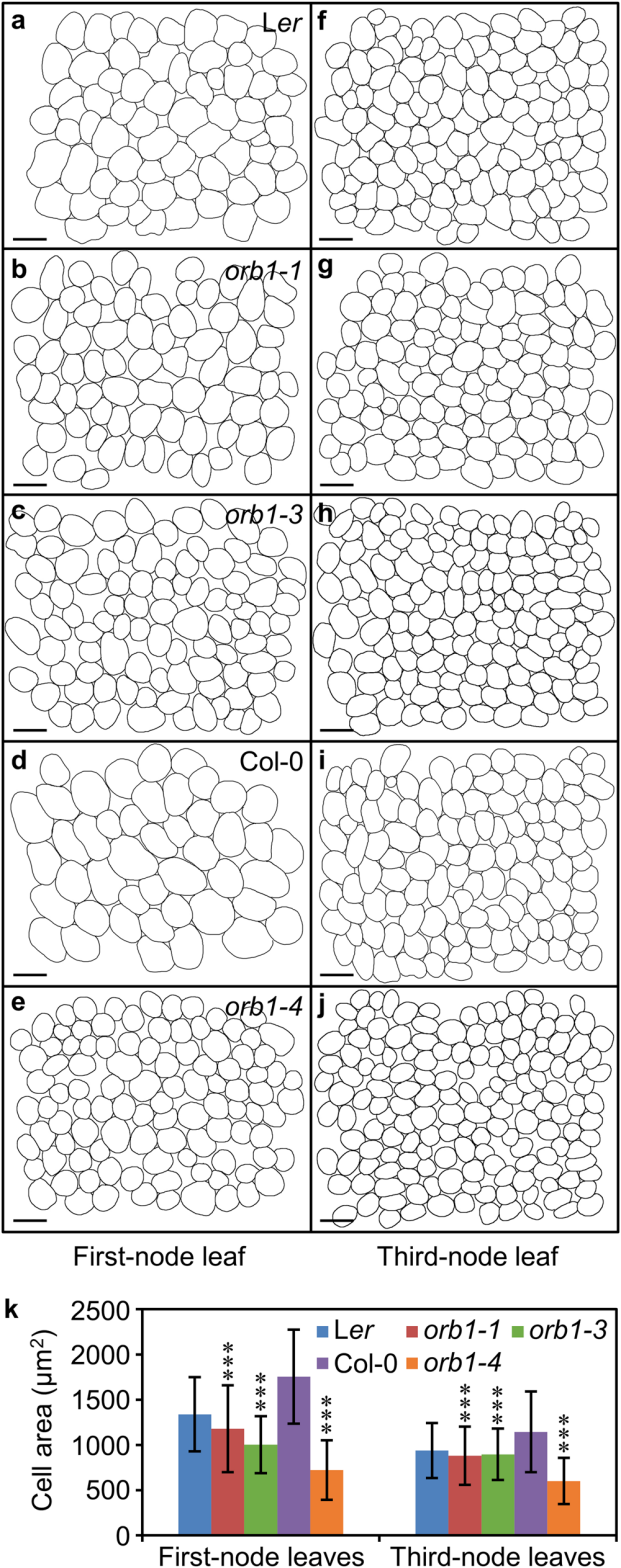
Morphometry of palisade mesophyll cells in *orb1* mutants. (**a–j**) Diagrams of the subepidermal layer of palisade mesophyll cells from (**a–e**) first- and (**f–j**) third-node leaves. (**k**) Palisade mesophyll cell area in first and third-node leaves. Plants were collected 21 das. Scale bars indicate 50 µm. Asterisks indicate values significantly different from the corresponding wild type in a Student’s *t*-test (***p < 0.001, n ≥ 600).

The pale green phenotype of *orb1* mutants was associated with significant reductions in the levels of chlorophyll a, chlorophyll b and carotenoids (p < 0.05; n = 5; Fig. 3), which were apparent in rosettes harvested between 12 and 24 das. The levels of chlorophyll a in *orb1-1*, *orb1-3* and *orb1-4* rosettes were, respectively, 47.7%, 63.4%, and 52.8% of those of the wild-type control plants. As regards chlorophyll b, its levels were, respectively, 45.8%, 57.9%, and 59.3% of those of the wild type. The carotenoid levels in *orb1-1*, *orb1-3* and *orb1-4* rosettes were, respectively, 55.9%, 74.4%, and 58.5% of those of the wild type (Fig. 4k).

We studied the internal tissues of *orb1* mutants using differential interference contrast (DIC) microscopy (Fig. 4). Rosette leaves from the first and third nodes were cleared using chloral hydrate. Two pictures per leaf were taken halfway along the primary vein and the leaf margin, using 6 leaves per genotype. Differences in the area of palisade mesophyll cells were tested using measurements from n ≥ 600 cells per genotype. A significant reduction of cell area was observed in palisade mesophyll cells using paradermal sections of leaves from the three mutants (p < 0.001; Fig. 4). The ratio of the area of leaf lamina to the area of palisade mesophyll cells, however, was similar in the mutants and the wild type, suggesting that the reduction in leaf size mainly results from the observed reduction in cell area.

### *ORB1* is the same gene as *Fd-GOGAT1*

**Figure 5:**
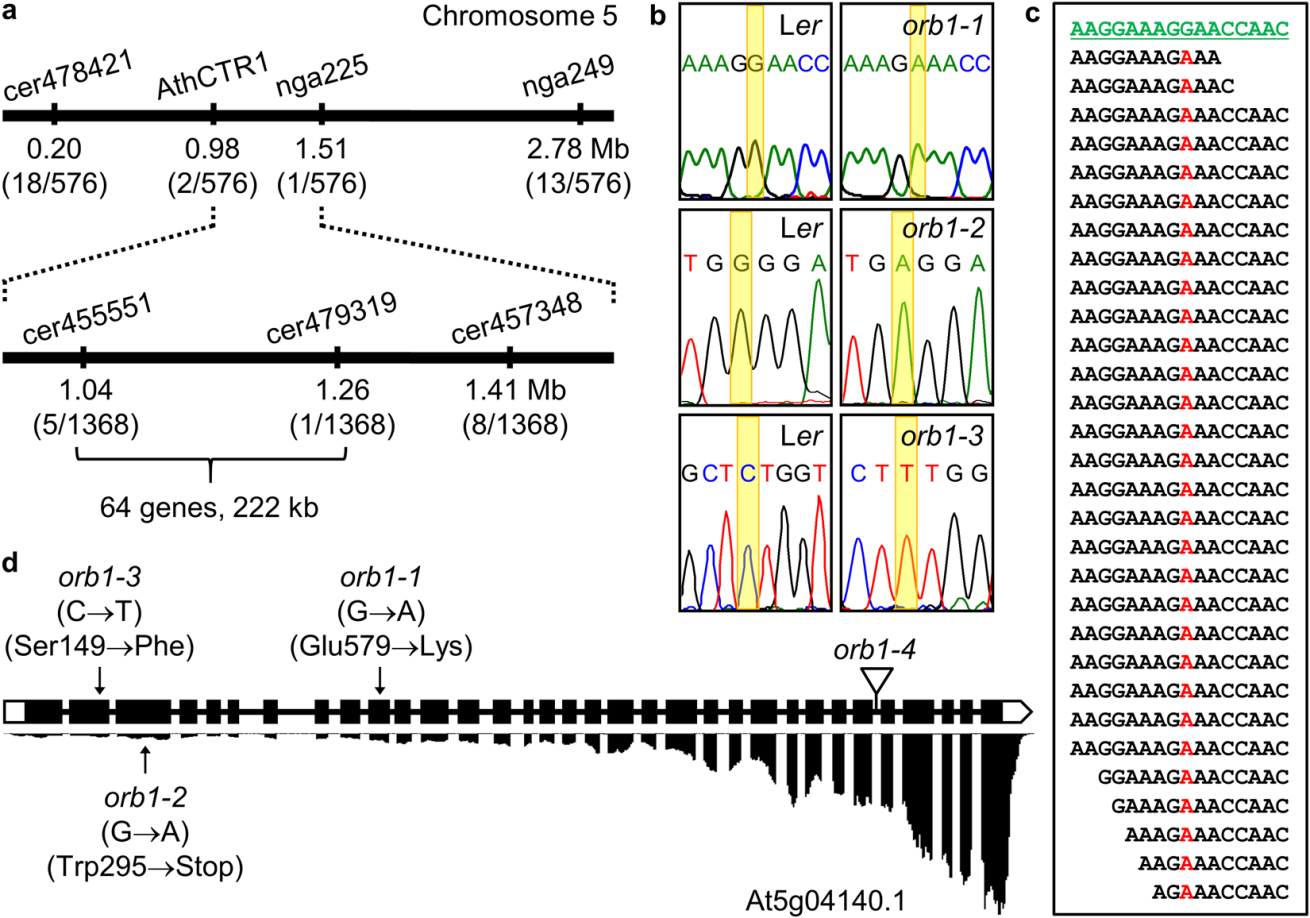
Positional cloning of the *ORB1* gene. (**a**) A mapping population of 684 F_2_ plants derived from an *orb1-1* × Col-0 cross allowed us to define a candidate interval of 222 kb on chromosome 5. Names and physical map positions of the molecular markers used for linkage analysis are shown. The number of recombinant chromosomes found and the total of chromosomes analyzed are indicated in parentheses. (**b**) Electropherograms showing the point mutations in the *orb1-1*, *orb1-2* and *orb1-3* mutants. (**c**) Pileup of reads derived from the *orb1-1* genome. The reference sequence is shown in green, and the *orb1-1* mutation is highlighted in red. (**d**) Structure of the At5g04140.1 isoform of the *ORB1* gene and alignment of RNA-seq read pairs to the At5g04140.1 isoform, with indication of the nature and position of the *orb1* mutations. Boxes and lines between boxes indicate exons and introns, respectively. White boxes represent the 5′- and 3′-UTRs. A triangle indicates the T-DNA insertion in *orb1-4*.

To clone the *ORB1* gene, we followed the approach outlined in Mateo-Bonmatí *et al*.^34^, with an initial high-resolution linkage mapping step followed by the resequencing of the complete genome. The *ORB1* gene was first mapped between the AthCTR1 marker and the telomere of chromosome 5 by analyzing simple sequence length polymorphic (SSLP) markers in an F_2_ mapping population derived from a cross involving the *orb1-1* mutant and wild-type Col-0 plants^35, 36^. By genotyping 324 F_2_ plants for additional SSLP and insertion/deletion (indel) markers using primers listed in Supplementary Table S1, we placed the mutation between the AthCTR1 and nga225 markers (Fig. 5a). The analysis of the cer455551, cer479319 and cer457348 markers, which are located between AthCTR1 and nga225, allowed us to map the position of the *orb1-1* mutation between cer455551 and cer479319, in a 222-kb candidate interval that encompasses 64 genes and is roughly delimited by the At5g03870 and At5g04440 genes (Fig. 5a). To identify the causal mutation within this candidate interval, we sequenced the complete *orb1-1* genome with the Illumina HiSeq2000 platform, using 25,864,186 read pairs. Of these, only 18,552,905 read pairs (71.73%) were concordantly aligned to the most recent version of the Arabidopsis nuclear genome then available (TAIR10)^37^ using Bowtie 2 (version 2.1.0)^38^, yielding a sequencing depth of 28.03×. After discarding the L*er*/Col-0 polymorphisms, we found that the *orb1-1* mutant carries a G→A transition mutation in the coding region of the At5g04140 gene (Fig. 5c). This mutation was confirmed by conventional Sanger sequencing (Fig. 5b). The At5g04140 gene encodes the chloroplast-localized FERREDOXIN-DEPENDENT GLUTAMINE OXOGLUTARATE AMINOTRANSFERASE 1 (Fd-GOGAT1) protein, also known as GLUTAMATE SYNTHASE 1 (GLU1)^39–41^. Although the TAIR10 annotation includes two different splice forms for this gene, At5g04140.1 (accession number NM_120496.3) and At5g04140.2 (NM_180432.2), we only obtained experimental evidence supporting the At5g04140.1 isoform, as shown by the alignment of RNA sequencing (RNA-seq) read pairs to the reference genome (see below; Fig. 5d). This isoform encompasses 33 exons, encoding a protein that is 1622 amino acids long and has a molecular mass of 176.9 kDa (Fig. 5d).

**Figure 6:**
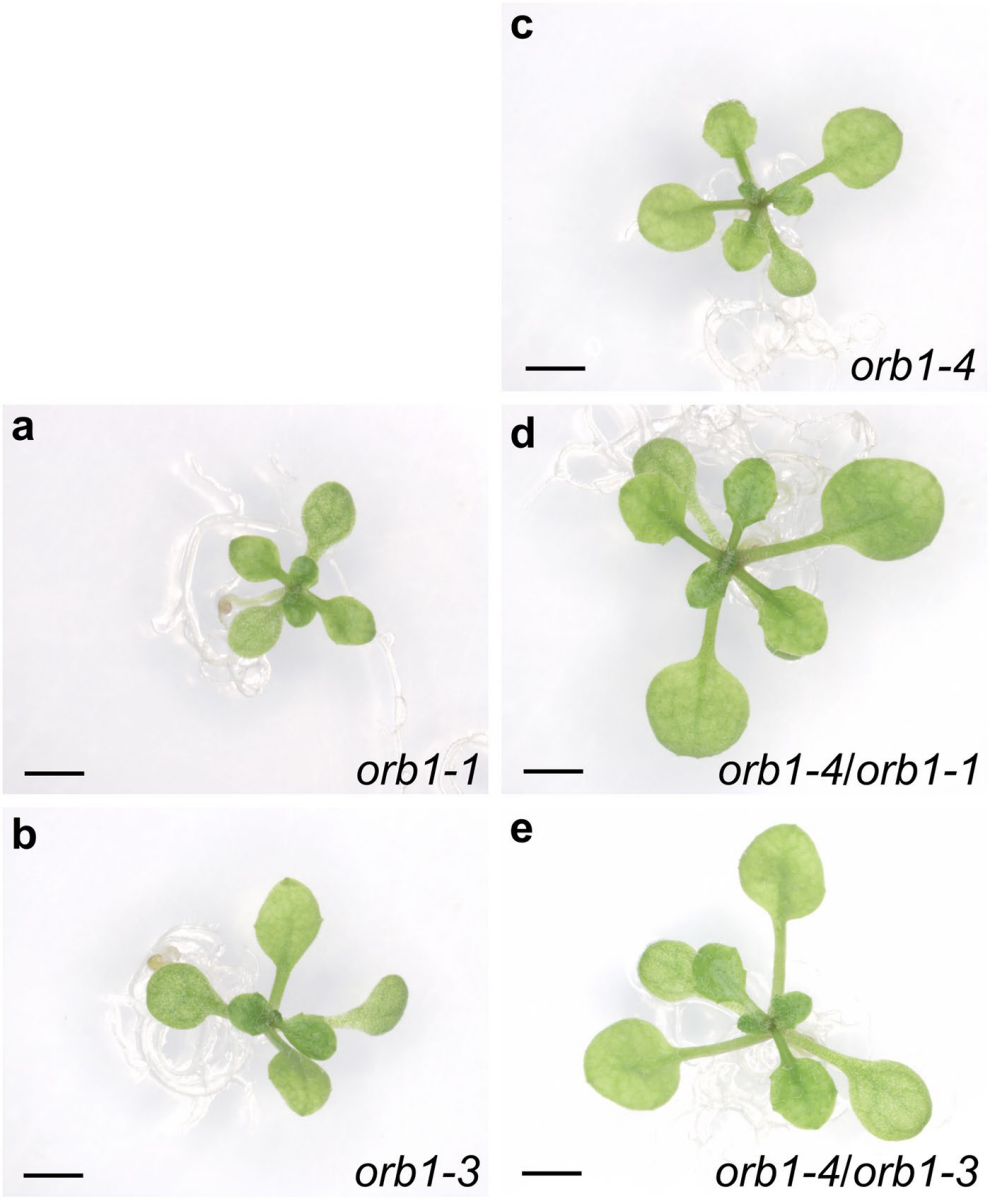
Allelism test of *orb1-1* and *orb1-3* point mutations with the insertional *orb1-4* mutation. Pictures were taken 14 das. Scale bars indicate 2 mm.

The ORB1 protein contains a glutamine amidotransferase type 2 domain (Class-II or type 2 GATase domain; IPR017932)^42^ and a glutamate synthase domain (IPR002932), which are connected by a glutamate synthase central-N domain (IPR006982). The glutamate synthase domain contains a putative flavin mononucleotide (FMN) binding site and a Fe-S cluster^43^. The hydrolysis of L-glutamine in the amidotransferase domain yields ammonium and L-glutamate. The ammonium is then combined with 2-oxoglutarate in the FMN binding domain to produce a second molecule of L-glutamate^44^. The G→A transition mutation found in the *orb1-1* mutant damages the first position of codon 579 of the At5g04140.1 coding sequence (exon 10), causing a lysine (K) for glutamate (E) substitution in the glutamate synthase central-N domain (Fig. 5c). A G→A transition mutation was found in the *orb1-2* mutant. This mutation alters the third position of codon 295, replacing a tryptophan (W) with a stop codon. A C→T transition mutation was found in the *orb1-3* mutant. This mutation damages the second position of codon 149 (exon 2) of the gene, causing a phenylalanine (F) for serine (S) substitution. The *orb1-2* and *orb1-3* mutations affect the glutamine amidotransferase type 2 domain. The T-DNA insertion of *orb1-4* is located in intron 28, according to a flanking sequence recovered from the left border of the T-DNA that is available from GenBank (accession number BH251122.1). Complementation crosses involving the EMS-induced *orb1-1* and *orb1-3* alleles as well as the insertional *orb1-4* allele showed that all these mutations damage the same gene (Fig. 6).

### Expression pattern of the *ORB1* gene

**Figure 7:**
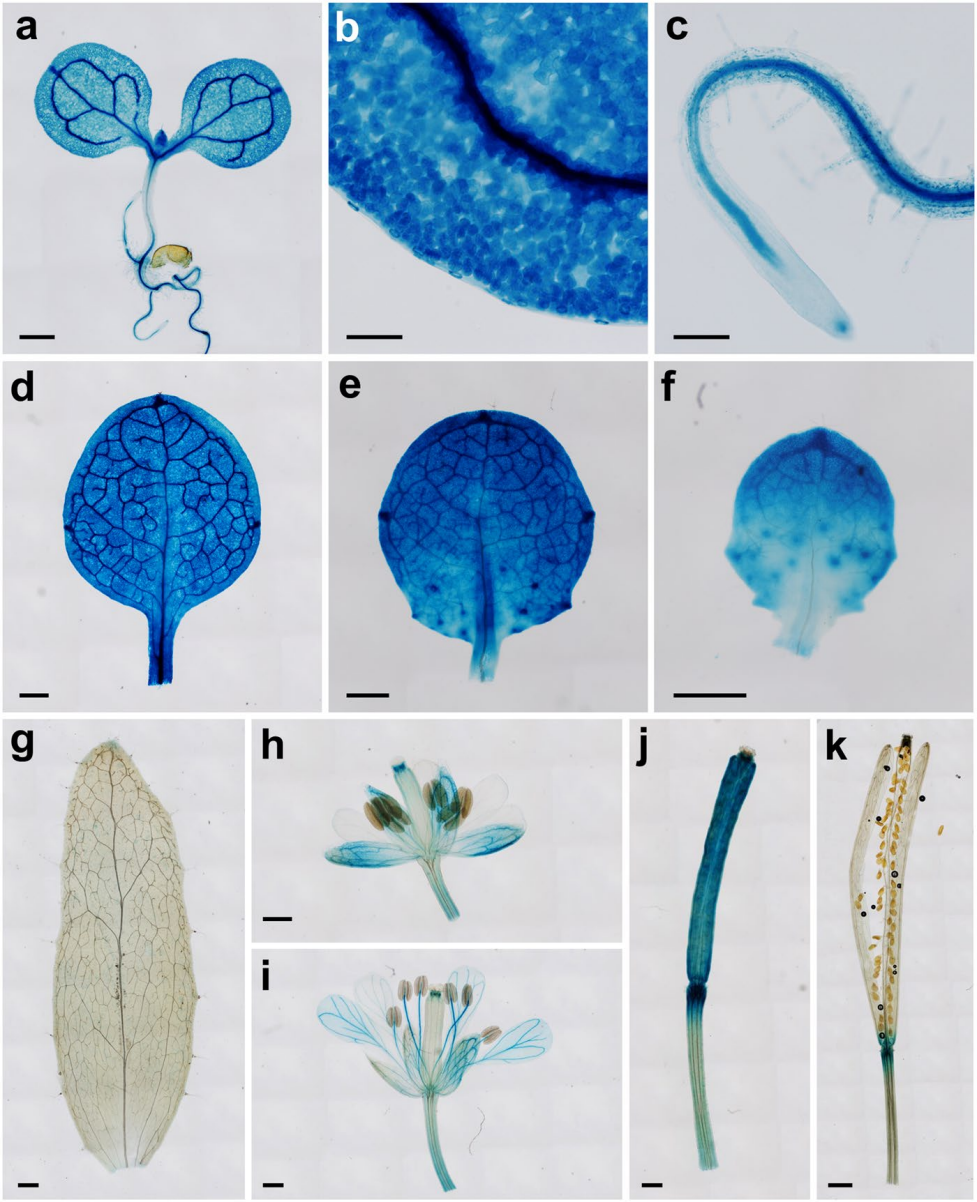
Visualization of *ORB1*_*pro*_ *:GUS* activity on a wild-type L*er* background. (**a**) Seedling, (**b**) cotyledon detail (vein and palisade mesophyll), (**c**) root, (**d**) first-node leaf, (**e**) third-node leaf, (**f**) fifth-node leaf, (**g**) cauline leaf, (**h**) immature flower, (**i**) mature flower, (**j**) immature silique, and (**k**) mature silique. Pictures were taken (**a–c**) 7, (**d–f**) 14, and (**g–k**) 40 das. Scale bars indicate (**k**) 1 mm, (**a,d–j**) 500 μm, and (**b,c**) 100 μm.

The Arabidopsis eFP Browser (http://www.bar.utoronto.ca/)^45^ and Transcriptome Variation Analyses (TraVA; http://travadb.org)^46^ databases indicate that the expression of *ORB1* is most intense in above-ground tissues. To define the expression pattern of *ORB1* more precisely, we generated an *ORB1*_*pro*_ *:GUS* construct. By transforming L*er* plants, we isolated two independent transformants expressing the *ORB1*_*pro*_ *:GUS* transgene. In young seedlings (7 das), we observed GUS signal in cotyledons, leaf primordia, shoot apical meristems and roots (Fig. 7a). In roots, the highest expression was detected at the vascular cylinder and root apices (Fig. 7c). In cotyledons and leaves, the GUS signal was most intense at the veins and the hydathodes, but mesophyll cells were also stained (Fig. 7b,d–f). This expression pattern matches the *GLU1* expression pattern previously described by other authors in tobacco and Arabidopsis^47, 48^. In cauline leaves, no expression of the transgene was observed (Fig. 7g). In immature flowers, we observed GUS staining at the sepals and the style (Fig. 7h). In mature flowers, GUS signal was detected at the anther filaments, the style and the venation pattern of petals and sepals (Fig. 7i). In immature siliques, GUS expression was intense (Fig. 7j), but in mature siliques we only observed GUS signal at the receptacle (Fig. 7k).

### RNA-seq analysis of the *orb1*–*3* mutant

To identify global changes in the transcriptome, we sequenced RNA samples isolated from *orb1-3* and Landsberg *erecta* (L*er*) vegetative rosettes, including three biological replicates of each genotype, using a strand-specific RNA-seq protocol. The reads were analysed using the Tophat2/Cufflinks pipeline^49–51^, indicating the expression levels for each gene as FPKM values (fragments per kilobase of transcript per million fragments mapped; Supplementary Table S2). In line with the results of a microarray analysis previously performed using a T-DNA allele of the same gene (*glu1-2*, SALK_019917)^33^, our results demonstrate that an extensive reprogramming of the transcriptome occurs in response to the *orb1-3* mutation. Out of 18856 different genes tested for differential expression levels, 6303 genes were found to be significantly differentially expressed between *orb1-3* and L*er* at a false discovery rate (FDR) threshold of 5%. Of these, 2833 genes were expressed at higher levels and 3470 genes at lower levels in *orb1-3* rosettes. When we used a stricter FDR threshold of 1%, 1862 genes and 2522 genes were respectively found to be expressed at higher and lower levels. We compared our results to the set of differentially expressed genes identified in the study of Kissen *et al*.^33^. Because the two studies differ in aspects such as ecotype (L*er* in this work versus Col-0 in that of Kissen *et al*.), choice of tissue (complete basal rosettes versus leaves), light regime (continuous lighting versus 16-hour photoperiod) and medium composition (half-strength versus full-strength MS salts), we expected to see differences in our results. A total of 1232 genes (28.1% of the 4384 differentially expressed genes selected at an FDR of 1%) were shared between the two studies, including 548 upregulated genes (29.4% of 1862) and 684 downregulated genes (25.6% of 2674). We expected that this set comprises genes whose expression reproducibly changes as a consequence of the *orb1* (*glu1*) mutations, regardless of other endogenous and environmental factors.

**Figure 8:**
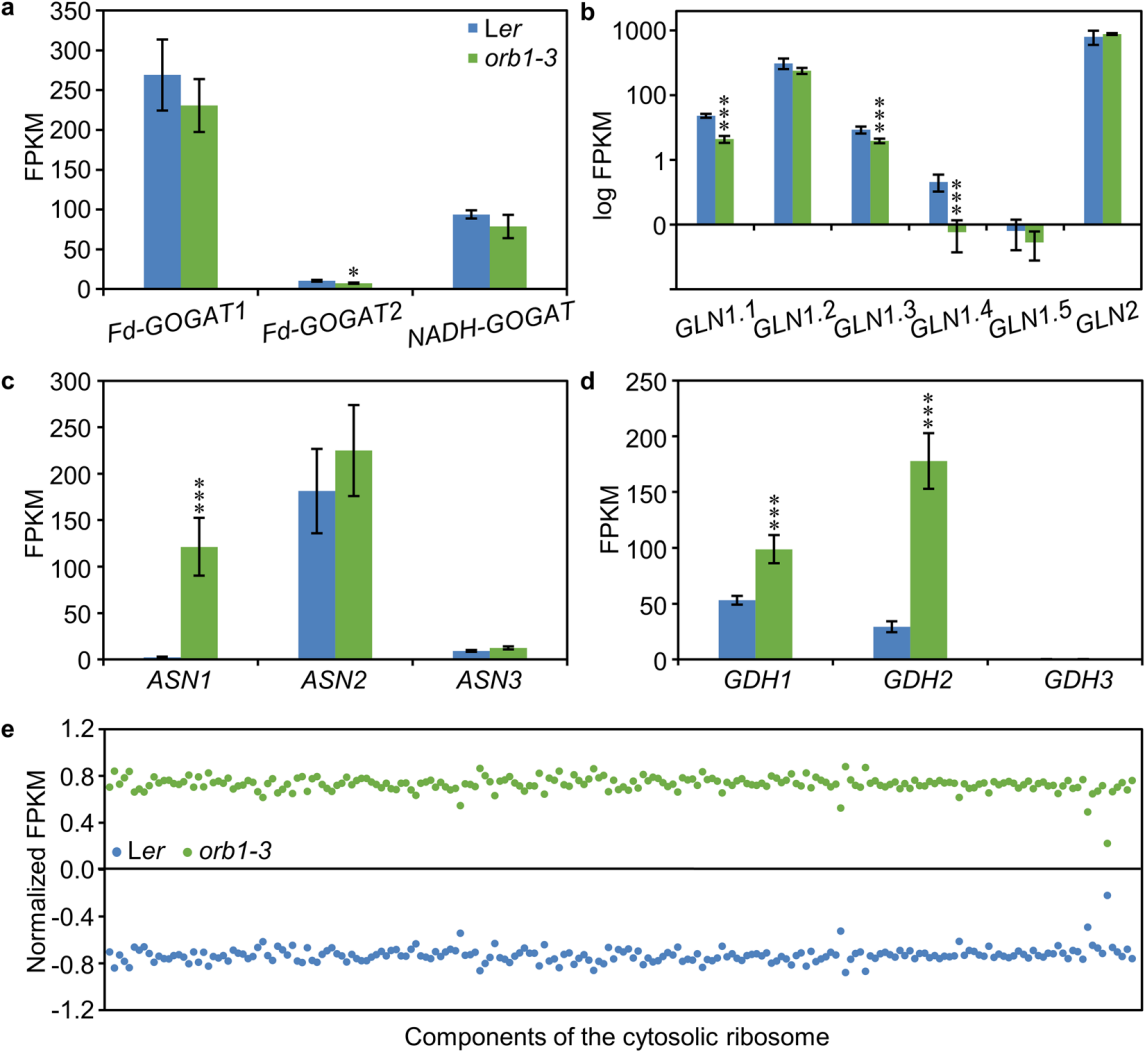
Differences in the expression of genes encoding glutamate-related enzymes and components of the cytosolic ribosome between L*er* and *orb1-3* plants. Expression levels of genes encoding (**a**) glutamate synthases (*Fd-GOGAT1*, *Fd-GOGAT2* and *NADH-GOGAT*), (**b**) glutamine synthetases (*GLN1.1*, *GLN1.2*, *GLN1.3*, *GLN1.4*, *GLN1.5* and *GLN2*), (**c**) asparagine synthetases (*ASN1*, *ASN2* and *ASN3*), (**d**) glutamate dehydrogenases (*GDH1*, *GDH2* and *GDH3*), and (**e**) several components of the cytosolic ribosome in L*er* and *orb1-3* plants, expressed as FPKM (fragments per kilobase of transcript per million fragments mapped). Error bars indicate standard deviations. Asterisks indicate values significantly different from L*er* as determined by Cuffdiff (*q < 0.05, ***q < 0.001, n = 3). The expression levels of the genes encoding components of the cytosolic ribosome were normalized based on the mean and standard deviation of the FPKM values obtained for each gene in all samples analyzed.

Different from the *glu1-2* mutant, which is a knock-down mutation^33^, our *orb1-3* point mutation did not significantly affect the abundance of its own transcripts (Fig. 8a), suggesting that the expression of *ORB1*/*Fd-GOGAT1* is not induced by glutamine, one of the reaction substrates, which is known to accumulate at increased levels in loss-of-function *glu1* mutants^28, 33, 52^. In contrast to previous results^33^, we found three genes encoding glutamine synthetases (At5g37600, At3g17820 and At5g16570) expressed at significantly reduced levels in *orb1-3* leaves, suggesting that the expression of these enzymes is subjected to product inhibition (Fig. 8b). We also found a drastic increase in the expression of *GLUTAMINE-DEPENDENT ASPARAGINE SYNTHASE 1* (*ASN1*, At3g47340), one of the three genes that encode glutamine-dependent asparagine synthetases in the Arabidopsis genome^53, 54^. Although *ASN1* was expressed at relatively low levels in the wild type, the *orb1-3* mutation caused a 60-fold increase in its expression (Fig. 8b). Asparagine synthetases, such as ASN1, transfer an amide group from glutamine to aspartate, yielding asparagine and glutamate. The expression of the genes encoding the small and large subunits of CARBAMOYL PHOSPHATE SYNTHETASE (CPS; *VEN3* and *VEN6*, respectively)^21^ was also found to be increased in *orb1-3* rosettes. Because CPS catalyzes the production of glutamate and carbamoyl phosphate from glutamine, the enhanced levels of CPS and ASN1 might help to compensate the lack of Fd-GOGAT activity by producing glutamate and by reducing the elevated glutamine levels. Asparagine can in turn be converted into different amino acids by the activity of *ALANINE:GLYOXYLATE AMINOTRANSFERASE 1* (*AGT1*, At2g13360), which encodes an asparagine aminotransferase in the Arabidopsis genome^55^, or into aspartic acid by the activity of two asparaginases, ASPARAGINASE A1 (ASPGA1, At5g08100) and ASPGB1 (At3g16150). Different from *ASN1*, the *AGT1* gene was expressed at high levels both in the wild type and the mutants (with FPKM values between 902 and 1235), suggesting that the conversion of Asn into Asp is not a limiting step. The expression of the two asparaginases was similarly not affected by the *orb1-3* mutation.

Two (At5g18170 and At5g07440) out of the three genes encoding glutamate dehydrogenases were expressed at elevated levels in the *orb1-3* mutant. In particular, the expression levels of *GLUTAMATE DEHYDROGENASE 2* (*GDH2*, At5g07440) shifted from 29 in Col-0 to 177 in the mutant (a fold change of ~6.1). Glutamate dehydrogenases convert glutamate into 2-oxoglutarate and hence, this increase in *GDH2* expression is at first sight unexpected because the increased GDH activity would further contribute to lowering the amount of glutamate. Counter-intuitively, the conversion of glutamate into 2-oxoglutarate might uncover a cellular strategy to duplicate the cellular pool of glutamate, as glutamate synthases such as Fd-GOGAT yield two glutamate molecules from each molecule of 2-oxoglutarate, in a reaction that also requires a molecule of glutamine.

To survey the metabolic pathways affected in the *orb1-3* mutant, we mapped the differentially expressed genes detected in our RNA-seq dataset using the KEGG PATHWAY online tool (http://www.genome.jp/kegg/pathway.html)^56, 57^. In addition to the above-described genes involved in nitrogen metabolism, one noticeable characteristic of the map was the opposite regulation of enzymes in the biosynthesis and degradation pathways of fatty acids. While most genes encoding enzymes involved in their biosynthesis were overexpressed (for instance At3g04000, At5g10160, At2g05990 and At2g30200), most genes involved in their degradation, such as At4g29010, were repressed (Supplementary Fig. S1).

As also indicated by the Gene Ontology (GO) analysis below, we found many genes encoding subunits of the cytosolic ribosome among the set of upregulated genes: as many as 208 genes displayed a concerted increase in their expression levels (Fig. 8e and Supplementary Fig. S2). Although this result remained hidden in the supplemental tables of Kissen *et al*., our results show that ribosomal proteins constitute the most abundant functional category among the genes shared by both studies. Protein biosynthesis is, undeniably, an important contributor to the nitrogen balance of the cell. Hence, this concerted upregulation possibly reflects a cellular response to altered amino acid levels when Fd-GOGAT1 function is impaired, and a concomitant altered translation.

### GO analysis and singular enrichment analysis of differentially expressed genes

We assigned GO terms to the complete set of genes differentially expressed between L*er* and *orb1-3* using the GO annotation of the Arabidopsis genome that is available from TAIR. The 4384 differentially expressed genes (at an FDR of 1%) were assigned a total of 1218 GO terms from the ‘molecular function’ subontology, 1771 terms from the ‘biological process’ subontology and 337 terms from the ‘cellular component’ subontology (up to a total of 3326 GO terms). We next considered the distribution of GO terms in the overexpressed and the underexpressed genes separately. The overexpressed genes were assigned 1113 ‘biological process’ terms, 269 ‘cellular component’ terms and 644 ‘molecular function’ terms. The set of underexpressed genes was assigned 1294 ‘biological process’ terms, 192 ‘cellular component’ terms and 124 ‘molecular function’ terms. A total of 636 terms (35.91%) from the ‘biological process’ subontology, 124 terms (36.8%) from the ‘cellular component’ subontology, and 350 terms (28.74%) from the ‘molecular function’ subontology were shared by the sets of underexpressed and overexpressed genes.

We performed singular enrichment analysis (SEA) for the GO terms assigned to the complete set of differentially expressed genes (Supplementary Table S3a). Thirty-three GO terms in the ‘cellular component’ subontology were significantly enriched. The most significantly enriched term was ‘cytosolic ribosome’ (GO:0022626), as 77.35% out of the 287 genes containing this term in the background set were differentially expressed. This term was followed by other highly significantly enriched terms related to ribosomes, such as ‘ribosomal subunit’ (GO:0033279), ‘ribosome’ (GO:0005840), ‘cytosolic large ribosomal subunit’ (GO:0022625), ‘large ribosomal subunit’ (GO:0015934), ‘cytosolic small ribosome subunit’ (GO:0022627), ‘small ribosomal subunit’ (GO:0015935), and ‘ribonucleoprotein complex’ GO:0030529). One hundred and eleven GO terms in the ‘biological process’ subontology were significantly enriched. In line with the most significantly enriched terms in the ‘cellular component’ subontology, the most significantly enriched term was ‘translation’ (GO:0006412), with 269 differentially expressed genes (45.98% out of the 585 genes containing this term in the background set). In line with previous results^33^, other enriched GO terms from this subontology were related to the responses to biotic and abiotic stimuli, including ‘defense response’ (GO:0006952), ‘response to biotic stimulus’ (GO:0009607), ‘response to stress’ (GO:0006950), ‘response to other organism’ (GO:0051707), ‘response to stimulus’ (GO:0050896), ‘response to wounding’ (GO:0009611), ‘response to external stimulus’ (GO:0009605), ‘response to chemical stimulus’ (GO:0042221), ‘response to bacterium’ (GO:0009617), ‘response to organic substance’ (GO:0010033), ‘response to fungus’ (GO:0009620), and ‘response to salicylic acid stimulus’ (GO:0009751). 30 GO terms were significantly enriched in the ‘molecular function’ subontology. The most significantly enriched term was ‘structural constituent of ribosome’ (GO:0003735), in agreement with the most significantly enriched terms in the ‘biological process’ and ‘cellular component’ subontologies. This GO term was ascribed to 227 different genes, out of the 327 genes that contained this term in the background set (69.42%). A variety of different catalytic and binding activities were also enriched in this set.

We also performed enrichment analysis on the sets of overexpressed and underexpressed genes separately (Supplementary Table S3b and S3c, respectively). For the overexpressed genes, we found 21 enriched GO terms in the ‘biological process’ subontology. Many of these terms matched the terms identified when the analysis was performed with the complete set of differentially expressed genes. At the top of the list were terms such as ‘translation’ (258 genes), ‘ribosome biogenesis’ (113 genes) and ‘ribonucleoprotein complex biogenesis’ (118 genes). These numbers closely matched the numbers obtained when all the differentially expressed genes were taken together, showing that the vast majority of the genes involved in translation and ribosome biogenesis have increased expression levels in the *orb1-3* mutant. This increase suggests that low amino acid levels compromise translation and trigger a cellular response aimed at compensating this defect. Such response would include a coordinated overexpression of most components of the large and small subunits of the cytosolic ribosome. Indeed, previous authors have found that genes encoding ribosomal proteins and other factors related to ribosome biogenesis are co-regulated by the p33^TCP20^ protein, which binds to the GCCCR motif present in their promoters, providing a mechanism to ensure their appropriate stoichiometry. This motif was also found in the promoter of the cyclin *CYCB1;1* gene, linking ribosome availability with cell division^58^. In line with our results, mutations in the *ARABIDOPSIS PUMILIO 23* (*APUM23*) gene, which encodes an RNA-binding domain protein that functions in rRNA processing and ribosome assembly, are also known to cause a similar overexpression of genes encoding ribosomal proteins^59^. We found 43 enriched GO terms in the ‘cellular component’ subontology, the most significantly overrepresented of which being ‘cytosolic ribosome’ (GO:0022626; p = 2.71e-143), followed by other terms related to ribosomal function. Eight GO terms were found to be enriched in the ‘molecular function’ subontology. The most significantly overrepresented term was ‘structural constituent of ribosome’ (GO:0003735). For the underexpressed genes, we found 133 enriched GO terms in the ‘biological process’ subontology, 50 in the ‘molecular function’ subontology, and 10 in the ‘cellular component’ subontology. The most significantly enriched terms in ‘biological process’ were ‘defense response’ (GO:0006952) and other terms related to responses to biotic and abiotic stimuli. The ‘molecular function’ terms included different binding and catalytic activities, as well as an important number of transcriptional regulators (246). The most significantly enriched terms in the ‘cellular component’ subontology were related to membranes.

We also performed SEA using the set of 1232 differentially expressed genes shared by both studies (Supplementary Figure S3). In this smaller set, terms related to translation and the function of ribosomes appeared enriched to a greater extent than in the broader set of 4384 differentially expressed genes selected at an FDR of 1%, discussed above (Supplementary Table S3). Unlike the terms related to ribosomes, numerous terms turned out to be not enriched when we considered the smaller set of differentially expressed genes shared by both studies. Lost terms included many related to the response to various types of biotic and abiotic stimuli, suggesting that the stress responses largely depend on the genetic background (i.e. they are accession-specific) or other external factors. The set of shared upregulated genes was also enriched in ribosome-related terms, and included some new terms that are not enriched when the upregulated and downregulated genes were taken together. Examples of such additional terms are ‘rRNA processing’ (GO:0006364) and ‘rRNA metabolic process’ (GO:0016072). In link with the pale green phenotype of the mutants, the set of shared downregulated genes was enriched in terms related to plastid function and photosynthesis, which were not enriched in the set of all the differentially expressed genes taken together.

## Concluding Remarks

Glutamic acid plays a central role in the assimilation of nitrogen and the biosynthesis of amino acids. Our results show that altered glutamate biosynthesis impairs plant growth and triggers a concerted transcriptional response that includes the upregulation of more than a hundred genes required for ribosome biogenesis. Understanding the relationship between protein synthesis and amino acid availability, and how they are regulated during plant growth and development, should help to engineer plants with increased biomass.

## Methods

### Plant material, growth conditions and crosses

Seeds of the Columbia-0 (Col-0) and Landsberg erecta (L*er*) wild-type accessions of *Arabidopsis thaliana* (L.) Heynh., as well as the T-DNA line SALK_011035 (N511035), were obtained from the Nottingham Arabidopsis Stock Centre (NASC). *orb1-1*, *orb1-2* and *orb1-3* mutants were isolated after a EMS-induced mutagenesis of L*er* seeds^26^. Plant culture and allelism tests were performed as reported in Berná *et al*. and Ponce *et al*.^26, 60^.

### Plant morphometry

Pictures and drawings from Arabidopsis rosettes, leaves and palisade mesophyll cells were obtained as previously described^61^. Rosette area and mesophyll cell area measurements were performed using the NIS Elements AR 3.1 image analysis package (Nikon).

### Fresh weight, dry weight and pigment concentration

Fresh and dry weight quantification, as well as pigment content determination of L*er*, *orb1-1*, *orb1-3*, Col-0 and *orb1-4* at different ages were performed as previously reported in Casanova-Sáez *et al*.^62^.

### Statistical analyses

We carried out statistical analyses to compare the phenotypic traits of *orb1-1*, *orb1-3* and *orb1-4* mutants with their corresponding wild-type plants. We used the Mann-Whitney U-test for 10 or less replicates and the Student’s *t*-test for more than 10 replicates.

### GUS staining

To induce GUS activity, plant tissues were incubated in 90% acetone for 10 min at −20 °C, and then transferred into GUS stain solution (2 mM 5-bromo-4-chloro-3-indolyl-β-glucuronic acid, 50 mM sodium phosphate, pH 7.2, 5 mM potassium ferrocyanide, 50 mM potassium ferricyanide, and 0.2% Triton X-100). Once in GUS stain solution, samples were infiltrated under vacuum for 20 min in the dark and then dark-incubated for 2 hours at 37 °C. GUS stain solution was removed and stained-tissues were cleared with 70% ethanol. Samples were mounted in an 8:2:1 (chloral hydrate:glycerol:water) solution and examined with a Nikon D-Eclipse C1 microscope.

### Identification of the *orb1-1* mutation

We combined map-based cloning and next-generation sequencing approaches to identify the gene affected by the *orb1-1*, *orb1-2* and *orb1-3* mutations. We performed the low-resolution mapping of the *orb1-1* mutation as described in Ponce *et al*.^35^. We used the SSLP and insertion/deletion (indel) markers cer478421, nga225, nga249, cer455551, cer479319 and cer457348 to reduce the candidate interval to a 222-kb region, by genotyping F_2_ plants derived from an *orb1-1* × Col-0 cross using PCR or Sanger sequencing. Primers used for fine-mapping of the At5g04140 gene are shown in Supplementary Table S1.

### Genome resequencing and RNA-seq

To identify the *orb1-1* mutation within the candidate region, we resequenced the genome of the *orb1-1* mutant at the Beijing Genomics Institute (BGI). Genomic DNA was isolated as described in Mateo-Bonmatí *et al*.^34^ and sequenced using the Illumina HiSeq2000 platform with 90 nt-long paired-end reads. The paired-end reads obtained from the resequencing of the *orb1-1* genome were aligned to the TAIR10 reference genome using Bowtie 2 (version 2.1.0)^38^. The resulting alignment was visualized using Tablet (version 1.15.09.01)^63^.

Total RNA was extracted from ~100 mg of L*er* and *orb1-3* rosettes collected 16 das using TRI Reagent (Sigma-Aldrich). Library construction and RNA sequencing was performed by StabVida (Caparica, Portugal), using 3 biological replicates per genotype. Libraries were prepared using the TruSeq Stranded mRNA Library Prep Kit (Illumina). Paired-end sequencing was performed by multiplexing the libraries in an Illumina HiSeq2500 system. The sequence alignment and the quantification of gene expression levels were performed as previously described in Mandel *et al*.^64^. Reads were aligned to the TAIR10 version of the Arabidopsis genome using TopHat v.2.0.12 and Bowtie2 v.2.1.0 with the following parameters: -p 8 (number of threads), supplying the annotation for the nuclear genes in a general feature format (GFF) file, and using default values for all other parameters. The resulting alignments were quantified with Cuffdiff v.2.2.1 masking the rRNA, tRNA, snRNA and snoRNA genes for quantification purposes. Differentially expressed genes were mapped to metabolic pathways using the KEGG PATHWAY online tool (http://www.genome.jp/kegg/pathway.html)^56, 57^.

SEA was performed using agriGO (http://bioinfo.cau.edu.cn/agriGO/)^65^ with the default options (statistical test: hypergeometric, multi-test adjustment method: Yekutieli, significance level: 0.01). The latest release of TAIR’s GO annotation was downloaded from TAIR on 13/02/2017. For the SEA, we separately used the sets of underexpressed and overexpressed genes as well as the complete list of differentially expressed genes as queries, including customized annotation data taken from TAIR^66^. As the customized annotated reference, we used the set of genes that had been tested for differential gene expression (marked “OK” in the output of Cufflinks^50, 67^).

### Constructs and plant transformation

A 1943 bp fragment containing the intergenic region between At5g04130 and At5g04140 was amplified from Col-0 genomic DNA using the Phusion polymerase (Thermo Scientific) and the ORB1-pro-F and ORB1-pro-R primers (which contain *att*B1 and *att*B2 sites; Supplementary Table S1). This fragment was cloned into pGEM-T Easy221 vector (provided by Prof. B. Scheres) using BP clonase II (Life technologies). The insert was transferred to pMDC163^68^ using LR clonase II (Life technologies) to generate the *ORB1*_*pro*_ *:GUS* construct. L*er* plants were transformed with the *Agrobacterium tumefaciens* strain C58C1 using the floral dip method^69^. T_1_ transgenic plants were selected on Petri dishes supplemented with 15 μg·ml^−1^ hygromycin B (Invitrogen).

## Electronic supplementary material

Supplementary Information

Supplementary Table S2

Supplementary Table S3
