# Supplementary material for: Deficient glutamate biosynthesis triggers a concerted upregulation of ribosomal protein genes in Arabidopsis
Source: Sci Rep. 2017 Jul 21;7:6164. doi: 10.1038/s41598-017-06335-4 (PMC5522406; doi:10.1038/s41598-017-06335-4)
Supplement: Supplementary file 1 — Supplementary Information [file 41598_2017_6335_MOESM1_ESM.pdf]

Deficient glutamate biosynthesis triggers a concerted upregulation of ribosomal protein genes

Tamara Muñoz-Nortes, José Manuel Pérez-Pérez, Raquel Sarmiento-Mañús,

Héctor Candela, and José Luis Micol

Supplementary Information

(Figures S1-S3, and Table S1)

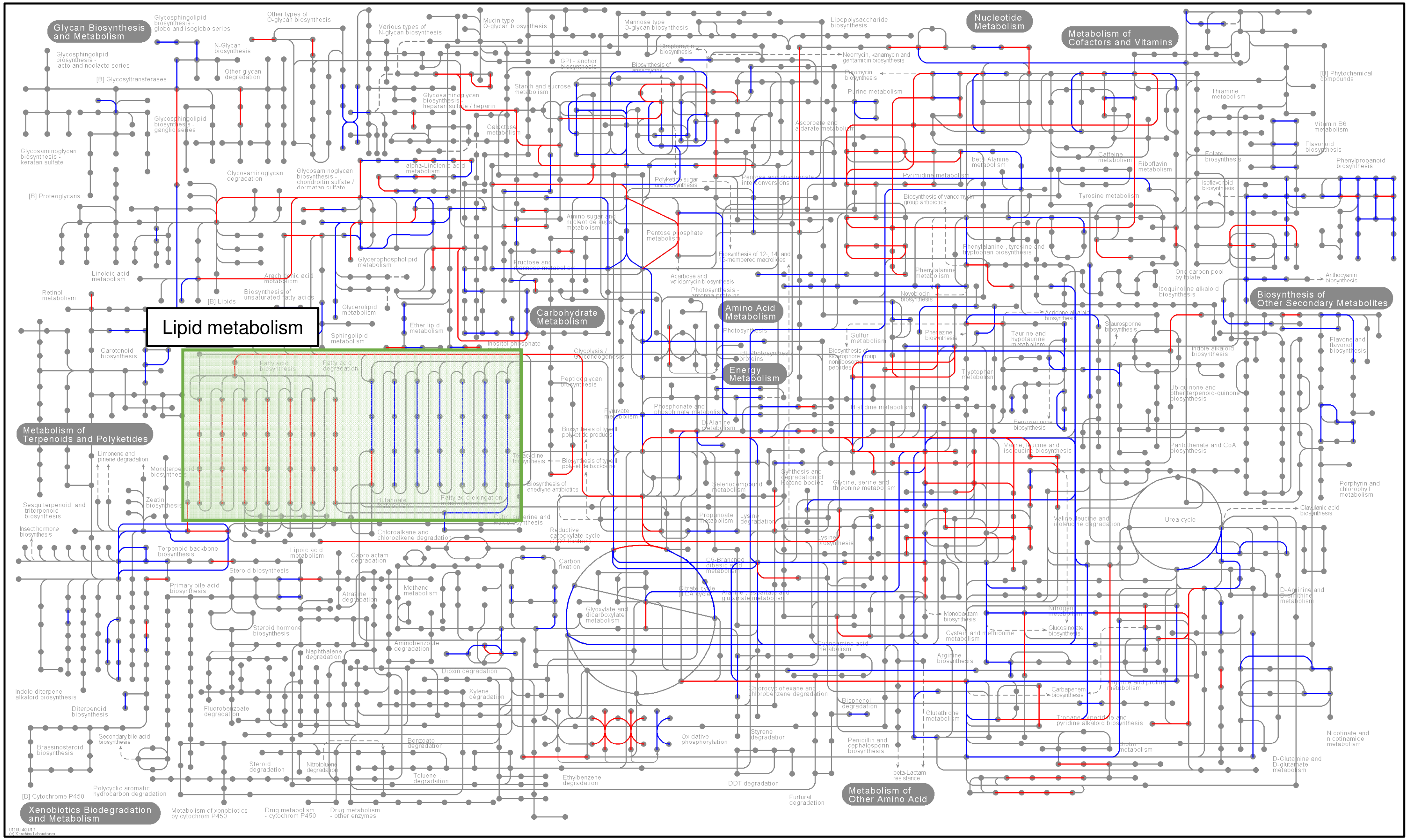

Supplementary Figure S1. Schematic view of metabolic pathways affected in the *orb1-3* mutant. The map was generated using the KEGG PATHWAY online tool [http://www.genome.jp/kegg/pathway.html; Kanehisa, M., and Goto, S. KEGG: Kyoto Encyclopedia of Genes and Genomes. *Nucleic Acids Res.* **28**, 27-30 (2000)]. Nodes represent metabolites and edges connecting adjacent nodes represent metabolic reactions. Grey nodes and edges represent reactions whose genes are not differentially expressed in our RNA-seq analysis. Upregulated and downregulated genes in *orb1-3* are shown in red and blue, respectively. The green rectangle highlights the fatty acid biosynthesis and degradation pathways.

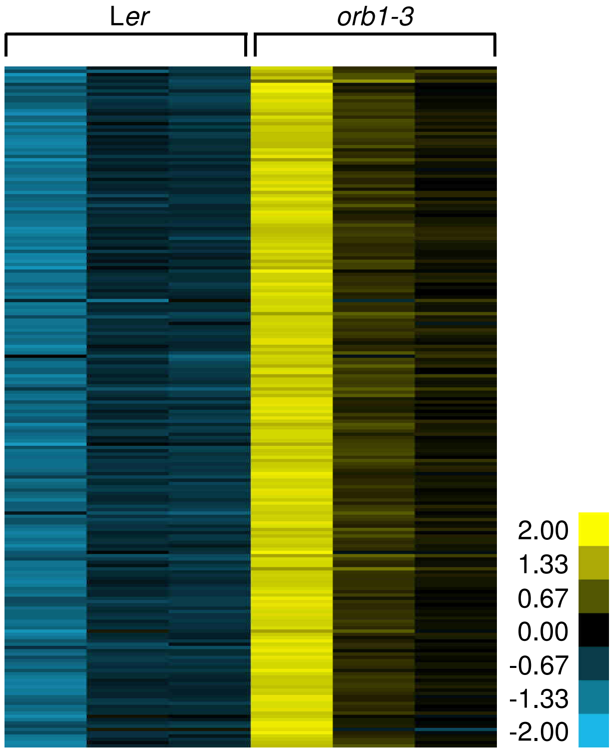

Supplementary Figure S2. Heat map showing the expression levels of genes encoding components of the cytosolic ribosome in L*er* and *orb1-3* plants. Each column represents the values of fragments per kilobase of transcript per million fragments mapped (FPKM) obtained from each RNA sample in an RNA-seq experiment. The expression levels were normalized based on the mean and standard deviation of the FPKM values obtained for each gene in the six samples analyzed (three L*er* and three *orb1-3*). The heat map was generated performing a hierarchical clustering of arrays with Cluster 3.0, and visualized using Java Treeview 3.0.

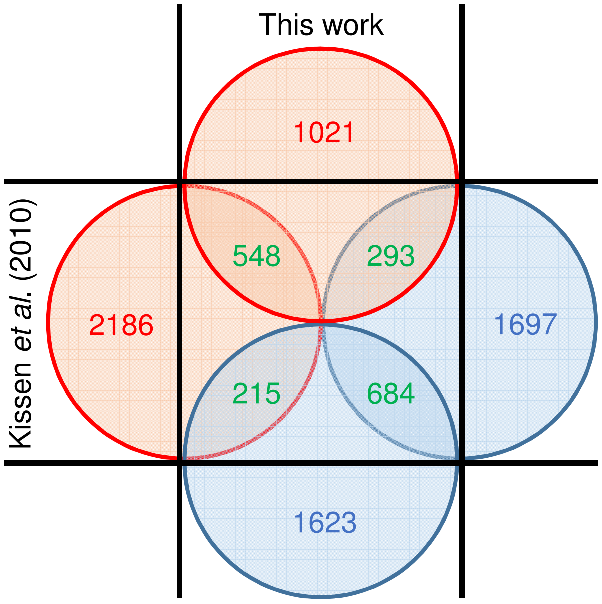

Supplementary Figure S3. Venn diagram showing similarities and differences between the microarray analysis performed by Kissen *et al.* (2010), and our RNA-seq analysis. Upregulated and downregulated genes are shown in red and blue, respectively. Common genes between both studies are shown in green.

Supplementary Table S1. Primers used in this work

Purpose	Oligonucleotide name(s)	Oligonucleotide sequences (5'→3')	
		Forward primer (F)	Reverse primer (R)
Linkage analysis	cer478421_F/R	CTCCAATTGCAGCCGAAACC	TGTAGTCCGAGAATCTAAAAATG
	AthCTR1_F/R	TATCAACAGAAACGCACCGAG	CCACTTGTTTCTCTCTCTAG
	cer455551_F/R	CATGCAAATTAGTTTACTGATTGCT	AAGCACACCTACAAGTCCGATTGA
	cer479319_F/R	TCAGAAGTCATTTGCAACTTGTAGA	CACAAATGATATATGGCTGTCGT
	cer457348_F/R	CTCACCACCCATCAGTTCATCT	CGGACGCAAATAAGTTTAATCGAGT
	nga225_F/R	GAAATCCAAATCCCAGAGAGG	TCTCCCCACTAGTTTTGTGTCC
	nga249_F/R	TACCGTCAATTTCATCGCC	GGATCCCTAACTGTAAAATCCC
Sequencing of candidate mutations	At5g04140_F1/R1	AGAAGCTCTCATCACTCATCTG	CTCAAATATGTTTTCAATAACTACA
	At5g04140_F2/R2	CTTGCTCCTTTTGATAAGTTG	CAGATGTTGTTACCTCAGGATAT
	At5g04140_F3/R3	TCATGATTAGAAGTGGAAGAAC	CTCATCAACAAACCTGGCCAT
	At5g04140_F4/R4	TCGGAGTTGTACCAGTTGATG	GACATTTTACCAGTCTATCGG
	At5g04140_F5/R5	CAAAGGTTCTGTCCACATATTTC	CCCACCTCATCAAAGGTGAGT
	At5g04140_F6/R6	TTATTGTGGTGCTCAGATATTTG	GATTGATCACCTGATGTAGATC
	At5g04140_F7/R7	AAGTTGCACAAGGTGCCAAGC	GCTGACCTCTTCTGCTACGTA
	At5g04140_F8/R8	CAGTGGGTGTAGCAAGTCAG	GCTAGTTTTACCTTTCCAACGT
	At5g04140_F9/R9	TGCAGGACAAGTGAACCTAAC	GCCACAACCTGCTCTTGAATG
	At5g04140_R10		GAATGTTGTTAGTTTATGTTAGAC
Gateway cloning	ORB1pro_F/R	GGGGACAAGTTTGTACAAAAAAGCAGGCT TCACCTTCACTGTGTAAAAGA	GGGGACCACTTTGTACAAGAAAGCTGGGT ATTGCATCGCCATTGAAATGA
Genotyping of bacterial clones and transgenic plants	pGEMT221_F/R	GTTGTAAAACGACGGCCAGTG	GGAAACAGCTATGACCATGATT
	pMDC163_F/R	CTCTAGCATTCGCCATTCAGG	ATTGCCCGGCTTTCTTGTAAC
